# Mapping the Multidimensional Link Between Spatiotemporal Gait Features and Metabolic Cost in Older Adults

**DOI:** 10.3390/s26144543

**Published:** 2026-07-17

**Authors:** Boyi Hu, Yuetong Wu, Xiangrui Wang, Amal A. Wanigatunga, Todd M. Manini

**Affiliations:** 1 Department of Industrial and Systems Engineering, University of Florida, Gainesville, FL 32611, USA; wuy2@ufl.edu (Y.W.); xiangruiwang@ufl.edu (X.W.); 2 Johns Hopkins Bloomberg School of Public Health, Johns Hopkins University, Baltimore, MD 21205, USA; awaniga1@jhu.edu; 3 Department of Health Outcomes & Biomedical Informatics and Institute on Aging, University of Florida, Gainesville, FL 32611, USA; tmanini@ufl.edu

**Keywords:** gait analysis, metabolic cost of walking, portable sensors, older adults

## Abstract

Walking becomes less economical with age, but the links between detailed gait mechanics and metabolic energy expenditure remain unclear. This study examined multidimensional associations between gait characteristics and walking energetics in community-dwelling older adults. Eighty-five participants, including 62.4% women, completed a steady-state walking task while metabolic energy expenditure was measured using portable indirect calorimetry. Four energetic outcomes were analyzed: walking cost as a percent of peak metabolic capability, steady-state walking metabolic rate, gross cost of transport, and reserve cost of transport. Gait was assessed using an instrumented walkway and summarized across spatial, temporal, and variability domains, yielding 28 gait parameters. Pearson correlations were calculated between each gait parameter and each metabolic outcome, with significance set at *p* < 0.05. Across 28 gait features and four metabolic outcomes, clear association patterns emerged. Spatial parameters showed the strongest and most consistent relationships with metabolic cost, suggesting that step length, stride length, and related forward-progression measures are closely tied to energetic demand during steady-state walking. Temporal parameters showed meaningful but generally weaker associations, while variability-based metrics also demonstrated moderate significant correlations. These findings provide a quantitative framework linking gait mechanics with walking energetics and may help identify mobility biomarkers and intervention targets in older adults.

## 1. Introduction

Population aging is accelerating worldwide, with the proportion of individuals aged 60 years or older projected to nearly double between 2015 and 2050 [1]. Mobility is central to independence and quality of life in late adulthood, yet age-related declines in gait and energy efficiency are pervasive. Approximately one-third of adults over 70 experience limitations in walking and balance that compromise daily activities [2,3]. Deterioration in gait is also a contributing factor for reduced physical activity and losses in social participation, thereby increasing the risk of disability [4]. Gait speed and its mechanics has been purported as a 6th vital sign because slowing is associated with morbidity, hospitalization, and all-cause mortality [5]. As a result, understanding the biomechanical and energetic factors associated with mobility decline has become a key priority for gerontological research and clinical intervention.

From a bioenergetic perspective, walking requires the continuous conversion of metabolic energy into mechanical work to sustain forward propulsion. Adults typically select a preferred walking speed that minimizes energy expenditure and maximizes efficiency [6]. With advancing age, both preferred speed and energetic efficiency decline, resulting in a higher metabolic cost during locomotion [7,8]. These increases have been linked to age-related reductions in muscle efficiency, altered gait mechanics, and compensatory control strategies [9]. Yet while the metabolic cost of walking and the mechanical characteristics of gait have each been extensively studied, they are rarely examined together in large samples of older adults. The extant of the literature has predominantly studied gait kinematics or kinetics in isolation, without concurrent measurement of metabolic demands. Conversely, as concluded by a recent report from the National Institute on Aging, there is a limited amount of knowledge that pairs metabolic investigations of walking economy with biomechanical contributors in robust samples [9].

As a result, the multidimensional associations between gait characteristics and the metabolic cost of walking remain incompletely understood, particularly in older adults. A small number of studies have attempted to link spatiotemporal parameters with energy expenditure, but most have relied on a small set of variables, low sample sizes and lack of low functioning older adults that preclude generalizable inferences [10]. Moreover, correlations between gait and metabolic metrics have not been studied comprehensively, leaving the overall pattern of associations unexamined. This fragmented literature has limited our ability to determine whether age-related gait characteristics, such as shorter step length, longer stance phase, or greater gait variability, are consistently associated with differences in walking energetic outcomes.

From a systems perspective, walking efficiency emerges from an interaction between neuromuscular control and physiological energy supply. With aging, the decline in both subsystems may produce a mismatch between motor execution and metabolic cost, leading to the characteristic slowing of gait with aging. Mapping the relationship between detailed gait features and metabolic outcomes can help identify which gait characteristics are most consistently associated with walking energetic performance. Identifying gait performance associated with relatively lower or higher metabolic demand may provide useful insight into mobility performance and help guide future investigations.

Recent advances in portable and sensor-based assessment have created new opportunities to quantify mobility outside of traditional motion-capture environments. Instrumented walkways such as GaitRite (CIR Systems, Franklin, NJ, USA) provide objective spatiotemporal gait measurements from pressure-sensing technology, while portable indirect calorimetry systems quantify oxygen consumption and energy expenditure during walking. Combining these complementary sensor-derived measures allows gait mechanics and physiological demand to be examined within the same mobility framework, which is directly relevant to sensor-based monitoring of human motion and functional decline in aging.

The purpose of this study was to explore the multidimensional association patterns between gait features and metabolic cost measures in a well-characterized cohort of older adults spanning a range of physical function levels. Using gait and metabolic datasets collected during standardized assessments within the same study, this study examined the relationships between spatiotemporal gait characteristics and multiple measures of walking metabolic cost. Rather than testing specific mechanistic hypotheses, the goal of this study was to establish a comprehensive empirical map of gait–metabolic associations across multiple gait domains and energetic outcomes.

## 2. Materials and Methods

### 2.1. Participants

**Figure 1:**
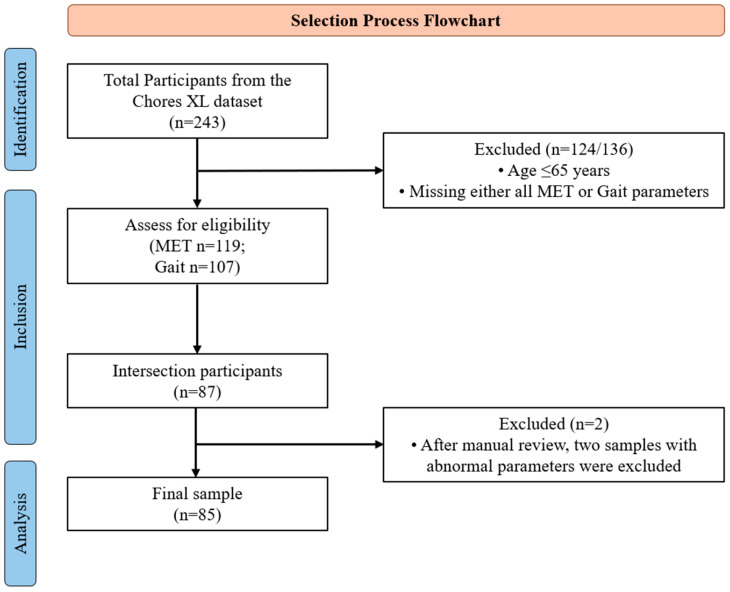
Selection process for older adults included in the current study.

Participants were community-dwelling older adults who took part in a laboratory-based study of walking biomechanics and energy cost [11]. For the present secondary analysis, we restricted the sample to individuals aged 65 years or older with valid measures of overground gait parameters and metabolic cost of walking. Among these, 119 participants had valid metabolic measurements, defined as having data available for at least one of the four metabolic outcomes (walking cost as a percent of peak metabolic capability, preferred walking metabolic rate, gross cost of transport, or reserve cost of transport). In parallel, gait data were available for 107 older adults from the instrumented walkway assessment. Gait availability was defined as having at least one of the 28 parameters. To construct the analytic sample for the present study, metabolic and gait datasets were merged. A total of 85 older adults had both metabolic and gait information (i.e., ≥1 metabolic parameter and ≥1 gait parameter) (Figure 1) and were therefore included in the final analysis.

### 2.2. Instrumentation, Experimental Protocol, and Outcome Measures

Portable indirect calorimetry was used to estimate energy expenditure during walking (COSMED K4b^2^ and K5, Rome, Italy). The instrumentation has been validated against Douglas bags during steady-state exercise [12]. Participants performed the standardized walking task continuously for approximately 8–10 min to achieve steady-state oxygen consumption. Oxygen consumption was measured breath-by-breath, smoothed using a 30-s running average, and steady-state VO_2_ was identified as an approximately 2-min plateau in oxygen consumption before the metabolic values were extracted. Prior to each testing session, the gas analyzers were calibrated using standard calibration gases (16.0% O_2_ and 5.0% CO_2_), room air calibration was performed, and the turbine flow meter was calibrated using a 3 L syringe. Steady-state oxygen consumption (VO_2_, mL/kg/min) was used as the primary estimate of “metabolic cost” of movement. Additionally, peak metabolic rate was determined from a graded treadmill test based on the modified Bruce Protocol [13]. Peak was defined as volitional failure, physically unable to continue the protocol, or reaching one of the following criteria: being within 10 bpm of age-predicted maximum, respiratory exchange ratio more than 1.10, or perceived exertion more than 9 on the Borg CR-10 scale.

Four metabolic outcomes were used in analyses: (1) M1-Walking cost as a percent of peak metabolic capability (%). (2) M2-Preferred-speed metabolic rate (mL/kg/min), which is steady-state VO_2_ at self-selected walking speed. (3) M3-Gross cost of walking, which is calculated as metabolic rate divided by walking speed (mL/kg/m). (4) M4-Metabolic Reserve or the amount of energy per meter remaining to walk additional distance.

Spatiotemporal gait metrics were obtained with an instrumented pressure-sensing walkway (GaitRite, CIR Systems, Franklin, NJ, USA), a platform with established validity for gait assessment. Gait features were not collected simultaneously with the above metabolic assessments, because the instrumented walkway and indirect calorimetry protocols served different measurement purposes and required different testing durations, so simultaneous collection was not feasible. The testing environment was the same between above VO_2_ and gait measurements. Each participant completed three traversals of the mat at a comfortable, self-selected speed with brief rests between passes. Participants were not permitted to use a cane or other type of walking aid during the test. For analysis, measures from the left and right limbs and from all trials were averaged to yield a single representative value per variable. As summarized in Table 1, outcomes were organized into spatial, temporal, and variability domains (G1–G28, 28 in total).

### 2.3. Statistical Analysis

In the first step, gait mechanics variables were screened using Pearson’s correlation coefficients (r) and their corresponding *p*-values (4 × 28 pairs in total). Pairwise correlations were computed using all available cases, the number of valid observations for each gait–metabolic pair varied due to missing data in some variability-based parameters. A small number of gait variables contained missing observations due to incomplete parameter generation in the original GaitRite output. No imputation was performed, and analyses were conducted using available-case data for each gait–metabolic pair. The resulting r and *p* matrices were organized into a cross-domain correlation map depicting the degree and direction of gait–metabolic associations. Given the large number of tests (112 correlations), we additionally applied the Benjamini–Hochberg false discovery rate (FDR) procedure to compute q-values and control for false positives. All tests were two-tailed, and statistical significance was set at *p* < 0.05. Correlation strength was interpreted as negligible (|r| < 0.30), low (0.30 ≤ |r| < 0.50) and moderate (0.50 ≤ |r| < 0.70) [14]. Given the substantial interrelationships among many spatiotemporal gait variables, the correlation analyses were intended as an exploratory characterization of multidimensional gait–metabolic association patterns rather than a series of independent confirmatory hypothesis tests. In addition to the correlation mapping, focused multivariable linear regression analyses were conducted to further characterize selected gait–metabolic relationships. The ten gait–metabolic pairs exhibiting the strongest correlation patterns were selected for follow-up multivariable regression analyses. For each selected gait feature, a separate regression model was constructed with a metabolic outcome as the dependent variable, adjusting for age, sex, and height. Standardized regression coefficients, 95% CI, and semi-partial R^2^ values were reported. All analyses were conducted using Python version 3.10.

## 3. Results

Among the 85 participants, 53 had SPPB scores ≥ 10, and 32 had SPPB scores < 10. These participants had a mean age of 73.7 ± 6.1 years, and the sample consisted of 53 females and 32 males. Average anthropometric characteristics were: weight 77.1 ± 16.9 kg, height 165.9 ± 9.2 cm, and body mass index (BMI) 27.9 ± 5.1 kg/m^2^ (Table 2). In addition, 35 participants (41%) exhibited peak aerobic capacity values below established functional thresholds (<18 mL/kg/min for women and <20 mL/kg/min for men), providing further context about the functional diversity of the cohort [15]. These characteristics indicate a typical community dwelling older adult cohort with substantial inter-individual variability in metabolic and gait performance.

**Figure 2:**
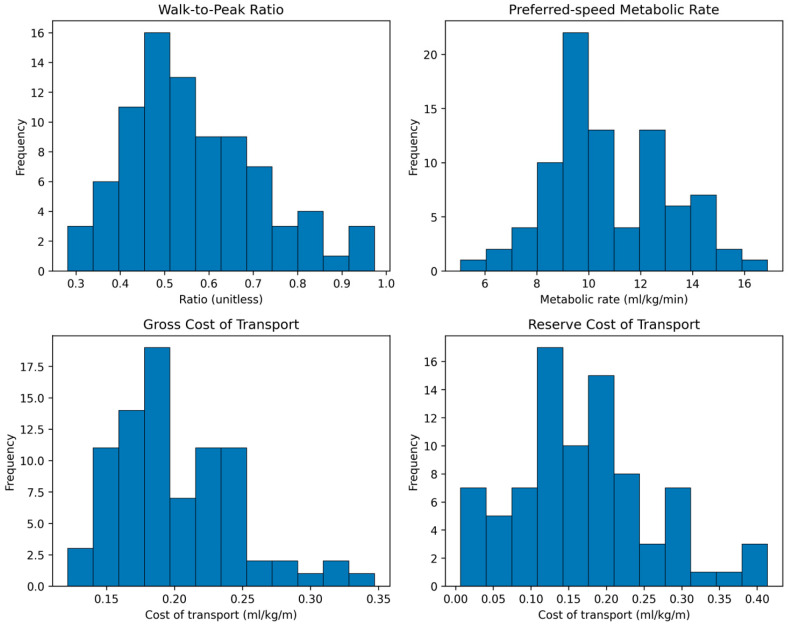
Distribution of metabolic outcomes among older adults.

Figure 2 shows the distributions of the four metabolic outcomes for the 85 older adults included in the analysis. All four measures display approximately unimodal distributions with sufficient spread across participants, indicating that the sample provides adequate variability in metabolic performance for examining gait–metabolism associations.

Table 3 and Table 4 summarize the metabolic outcomes and gait features for the full sample and stratified by physical function level based on SPPB. In general, participants with higher physical function (SPPB ≥ 10) exhibited higher preferred-speed metabolic rate compared to those with lower function, whereas gross and reserve cost of transport showed smaller between-group differences.

**Figure 3:**
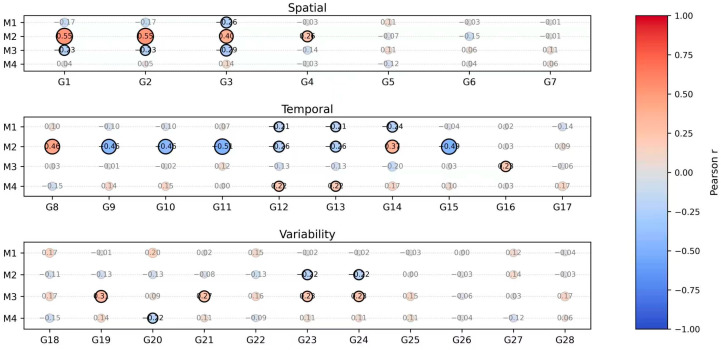
Correlation matrix between metabolic cost measures (M1 to M4) and gait parameters (G1 to G28). Bubble color reflects the strength and direction of correlation (blue = negative, red = positive). Bubble size is proportional to the absolute magnitude of the correlation coefficient. Opacity indicates statistical significance: non-transparent bubbles represent significant correlations, while transparent bubbles represent non-significant correlations.

**Figure 4:**
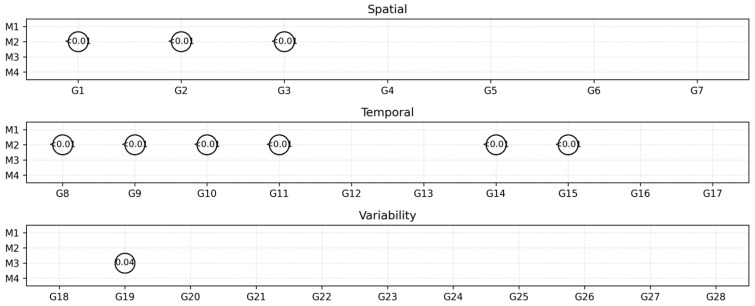
FDR-corrected associations between gait features and metabolic cost outcomes.

Spatial gait features showed the strongest and most consistent associations with metabolic outcomes (Figure 3 and Figure 4, Table S1). Measures reflecting forward progression, including total walking velocity (G1) and stride velocity (G2), were moderately and positively correlated with preferred-speed metabolic rate (M2) (r ≈ 0.50–0.60, *p* < 0.001). These relationships remained statistically significant after FDR correction (q < 0.05), indicating that individuals who walked faster exhibited higher absolute metabolic power. In contrast, correlations between velocity measures and distance-normalized energetic outcomes, gross and reserve cost of transport (M3–M4), were small and not statistically significant after FDR adjustment.

Step length (G3) demonstrated similar but slightly weaker patterns, showing positive associations with M2 that was statistically significant at the unadjusted level and, for several comparisons, survived FDR correction. No meaningful relationships were observed between step or stride length and cost-of-transport measures. Other spatial parameters, including base of support (G5), toe-out angle (G6), and step-length differential (G7), exhibited uniformly weak correlations with all metabolic outcomes and did not reach significance after FDR correction. Overall, spatial features associated with forward progression accounted for the largest proportion of variance in metabolic power, whereas lateral and asymmetry-related spatial measures contributed minimally.

Temporal gait parameters also showed systematic associations with metabolic cost, although effect sizes were generally smaller than those observed for spatial features and were primarily evident for preferred-speed metabolic rate (M2). Higher cadence (G8) and shorter temporal intervals, including cycle time, step time, double support time, and stance time (G9–G11, G15), were consistently associated with higher preferred-speed metabolic rate, with correlation magnitudes ranging from small to moderate (|r| ≈ 0.30–0.55). Several of these associations, particularly those involving cadence and overall cycle timing, remained statistically significant after FDR correction.

Heel-off timing (G14) showed one of the strongest temporal relationships with absolute metabolic demand, demonstrating a robust positive correlation with M2 that survived FDR adjustment. In contrast, associations between temporal parameters and the walk-to-peak ratio (M1) were generally weak and did not retain significance after multiple-comparison correction, suggesting that relative energetic demand normalized to aerobic capacity is less tightly coupled to gait timing characteristics.

Single support time and swing time (G12–G13) were only weakly related to metabolic outcomes and did not retain significance after FDR correction. Temporal asymmetry measures exhibited limited influence: step time differential (G16) showed a modest unadjusted association with gross cost of transport (M3), but this relationship did not persist after FDR adjustment, while cycle time differential (G17) was largely unrelated to all metabolic measures. Collectively, these findings indicate that global temporal characteristics governing gait rhythm are linked to absolute metabolic demand during walking, whereas finer-grained temporal asymmetries and fitness-normalized energetic measures show less robust coupling.

Variability-based gait metrics exhibited more modest and heterogeneous associations with metabolic outcomes compared with spatial and temporal features. Greater variability in step and stride characteristics, including step length SD, step time SD, stride length SD, stride time SD, and stance time SD (G18–G21, G25), showed selective associations with walking economy, primarily reflected by positive correlations with gross cost of transport (M3) and negative correlations with reserve cost of transport (M4).

However, only a subset of these variability-related associations remained statistically significant after FDR correction, indicating that while gait variability is related to energetic efficiency; its influence is weaker and less consistent than that of mean spatiotemporal gait characteristics.

Furthermore, multivariable linear regression models were conducted for ten selected gait–metabolic pairs to evaluate whether gait features exhibited independent associations with metabolic outcomes after adjusting for age, sex, and height. Table 5 summarizes standardized regression coefficients (β), *p*-values, and semi-partial R^2^ values for each model. Spatial parameters showed the strongest independent effects. For instance, total velocity (G1) and stride velocity (G2) remained a significant predictor of preferred-speed metabolic rate (M2), with the largest standardized coefficient and semi-partial R^2^, indicating substantial unique variance explained beyond demographic covariates. Temporal features related to gait rhythm, such as cadence (G8), cycle time (G9), step time (G10), and double support time (G11), also showed significant independent contributions to preferred-speed metabolic rate (M2), although effect sizes were smaller than those of spatial parameters. Heel-off timing (G14) remained a statistically significant predictor of preferred-speed metabolic rate (M2) after adjustment, consistent with its strong bivariate correlation. Step time variability (G19) also demonstrated a significant relationship with gross cost of transport (M3), indicating that fluctuations in step timing contribute meaningfully to the energetic demands of walking.

## 4. Discussion

The primary contribution of this study is the comprehensive characterization of multidimensional associations between spatiotemporal gait characteristics and multiple measures of walking metabolic cost in older adults. Unlike previous studies that typically focused on a limited number of gait variables or energetic outcomes, the present work provides a broader association map spanning multiple gait domains and metabolic measures within a heterogeneous cohort of older adults. Across 28 gait parameters and four metabolic outcomes, a clear and consistent pattern emerged: spatial velocity-related measures and global temporal rhythm were the strongest and most robust determinants of walking energy expenditure, whereas most variability metrics showed weaker or less consistent associations. These patterns were detectable in part because our cohort was relatively large and functionally diverse, it is noteworthy that the cohort exhibited substantial heterogeneity in aerobic capacity: 41% of participants fell below sex specific functional VO_2_peak thresholds (<18 mL/kg/min for women; <20 mL/kg/min for men) (Table 2). This broad metabolic range, together with the inclusion of both high and low functioning older adults, likely enhanced our ability to detect meaningful gait–metabolism associations that may be obscured in smaller or more homogeneous samples.

Spatial gait characteristics demonstrated the most robust relationships with metabolic outcomes, in line with prior work linking walking speed and stride mechanics to energetic demand [16]. In our dataset of older adults, both walking velocity and stride velocity were positively associated with preferred-speed metabolic rates. This pattern is consistent with previous findings demonstrating that a preferred walking speed that minimizes the cost of transport and that deviations (either increase or decrease) from this optimum increase metabolic cost [17]. Mechanical and modeling studies further support the idea that combinations of walking speed and step length largely determine the mechanical work of step-to-step transitions and, in turn, metabolic expenditure during transport [18]. The associations we observed between step length and metabolic cost are also broadly consistent with previous reports in aging populations. Older adults generally show higher metabolic cost of walking than young adults [19], and studies have linked slower gait and shorter steps to elevated energetic cost and reduced functional capacity [20]. In our data, longer step length and higher stride velocity were associated with higher preferred-speed metabolic rate. Their associations with gross cost of transport were weaker and did not remain significant after FDR correction. Thus, the apparent lower distance-normalized cost in faster walkers should be interpreted as an exploratory trend. At the same time, not all spatial parameters were strongly related to metabolism. Base of support and foot progression angle showed minimal associations with any metabolic outcome, which aligns with evidence that frontal–plane balance strategies can change markedly with age and instability without necessarily exerting a primary influence on metabolic cost [21]. Consistent with prior work, parameters related to frontal–plane balance or foot placement (e.g., base of support, foot progression angle) showed minimal associations with metabolic outcomes. Although these measures did differ between the high- and low-function groups (Table 4), these differences did not translate into meaningful variation in metabolic cost. This pattern suggests that frontal–plane adaptations may be more indicative of functional or balance-related limitations than of the energetic demand of walking.

Temporal gait parameters showed moderate, but consistent associations with metabolic cost of walking. Shorter cycle times and stride times were associated with higher metabolic rate, likely reflecting the increased cadence and muscular activation required to sustain faster rhythmic patterns. This is consistent with prior evidence that cadence driven increases in step frequency elevate the metabolic cost of transport, even when walking speed is held constant [22,23]. Heel-off timing showed one of the strongest temporal associations with metabolic rates. Prior work indicates that earlier heel-off in older adults does not necessarily reflect stronger ankle push-off capacity. Instead, it often arises from reduced plantar flexor power and a compensatory reliance on hip flexor activity to initiate limb advancement [24,25]. Such proximal compensation is metabolically more demanding and has been widely observed as part of the distal-to-proximal redistribution characteristic of aging gait [26]. Other temporal features related to balance control, such as single support time, swing time, and stance time, demonstrated weaker or no associations with metabolic outcomes of walking [27].

Variability-based gait parameters exhibited more modest and less consistent associations with metabolic outcomes compared with spatial and temporal features. Among the variability metrics, step time variability (G19) showed the clearest relationship, demonstrating a small but statistically significant association with gross cost of transport (M3). This pattern is broadly consistent with experimental and conceptual work showing that increased temporal variability can elevate neuromotor control demands and, in some contexts, increase metabolic cost [28,29,30]. One plausible interpretation is that greater stride-to-stride inconsistency requires more frequent corrective adjustments, which may modestly increase energetic demand. However, most variability measures, including variability in step length, stride length, swing time, and stance time, did not show meaningful associations with metabolic outcomes in this cohort. These findings suggest that gait inconsistency is not uniformly linked to energetic cost. Variability in frontal–plane measures showed minimal associations with any metabolic parameter, further indicating that sagittal-plane rhythm variability may be more mechanistically relevant to metabolic demand than other forms of gait variability.

A key contribution of this work is its comprehensive examination of gait–metabolic associations across a broad set of parameters within the same cohort. Previous studies have typically focused on a limited number of gait metrics or have examined metabolic cost in isolation. By characterizing associations across spatial, temporal, and variability domains simultaneously, this study provides a detailed and integrated view of how different aspects of gait mechanics contribute to energetic demand. This multidimensional mapping offers a useful empirical foundation for future modeling efforts, intervention development, and targeted rehabilitation strategies for older adults. Although correlation magnitudes ranged from approximately 0.20 to 0.60, such values are expected given the multifactorial nature of metabolic cost. Energetic expenditure reflects the integration of muscular physiology, neuromotor control, biomechanics, balance, and individual fitness, making it unlikely that any single gait parameter would account for a large proportion of variance. From a sensor-based assessment perspective, this finding is important because it suggests that portable gait and metabolic sensing systems provide complementary information about mobility in older adults. GaitRite-derived gait features primarily captured how participants organized movement across space and time, whereas portable indirect calorimetry quantified the physiological cost required to produce that movement. Therefore, gait sensor outputs should not be interpreted as direct substitutes for metabolic measurements, but rather as movement-based indicators that help explain variation in walking energy demand. This distinction is important for future sensor-based monitoring approaches that aim to identify older adults with inefficient or declining mobility.

Across the four metabolic outcomes, we observed a consistent pattern in which preferred-speed metabolic rate (M2) showed the largest and most coherent set of associations with gait, whereas the walk-to-peak ratio (M1) and the two cost-of-transport measures (M3–M4) exhibited notably weaker relationships. This attenuation is expected given that M1, M3, and M4 are all normalized or composite indices. The walk-to-peak ratio (M1) expresses walking VO_2_ relative to aerobic capacity, effectively removing variance shared with overall fitness and reducing its direct coupling to gait mechanics. Likewise, gross and reserve cost of transport (M3–M4) are normalized by walking speed, which mathematically removes much of the variance driven by spatial and temporal gait parameters and yields a narrower inter-individual range. In addition, although FDR correction was applied to reduce false-positive findings, many spatiotemporal gait variables are mathematically and physiologically interrelated. Therefore, some associations that were statistically significant before correction but did not survive FDR adjustment may still represent exploratory signals, particularly when they occur within coherent gait domains. These findings should be interpreted cautiously and require confirmation in future studies.

To complement the correlation mapping, multivariable linear regression analyses were conducted to evaluate whether the most prominent gait–metabolic pairs persisted after adjusting for age, sex, and height. These focused models revealed a clear and consistent pattern: only a small subset of gait parameters, primarily those reflecting overall gait rhythm (e.g., step time variability–G19) and temporal coordination (e.g., cycle time, cadence), retained independent associations with metabolic outcomes. The magnitude of the independent effects was modest, with standardized β values generally in the small-to-medium range and semi-partial R^2^ values indicating limited unique contributions to metabolic variance. Taken together, these results suggest that while certain gait characteristics are reliably associated with metabolic demand, much of their shared variance overlaps with age, sex, height, or overall walking behavior, reinforcing the importance of interpreting simple correlations within a broader health and aging context.

There are several strengths and limitations of this study. First, relative to other work in the field, this study included a large sample, collected peak oxygen consumption, and intentionally designed an inclusion criterion to ensure older adults with lower physical function were represented. Additionally, prior work has focused on a limited number of gait metrics or have examined metabolic cost in isolation (i.e., comparing age differences). For instance, characterizing spatial, temporal, and variability domains together gives a clearer, more integrated picture of how gait mechanics shape metabolic demands of walking in late-life.

As for limitations, the work used an observational cross-sectional design that precludes causal inference and may not reflect a direct mechanistic relationship. Gait variability measures are generally more sensitive to the number of recorded steps than mean spatiotemporal parameters. Therefore, the relatively small number of recorded steps in the present experiment may have limited the stability of the variability estimates and reduced the ability to detect associations with metabolic outcomes. An additional limitation is that gait and metabolic assessments were not performed simultaneously. Therefore, the observed relationships should be interpreted as associations between stable participant-level gait characteristics and metabolic outcomes rather than direct biomechanical–energetic interactions measured during the same walking task. The GaitRite instrumentation is also limited, no joint kinetics or muscle-level factors were collected. Furthermore, participants requiring walking aids were excluded from the study. Therefore, the present findings may not generalize to older adults with more severe mobility impairments or those requiring walking assistance.

## 5. Conclusions

In this study, we systematically examined associations between 28 spatiotemporal gait characteristics and 4 metabolic outcomes in a cohort of older adults. Velocity-related gait measures demonstrated the strongest and most consistent associations with preferred-speed metabolic rate, whereas gait variability measures generally exhibited weaker relationships. Multivariable regression analyses further indicated that individual gait features explained a modest but meaningful proportion of the variance in metabolic outcomes, with total velocity emerging as the strongest independent predictor.

Overall, the findings suggest that walking energetics in older adults are more strongly associated with gait speed and temporal gait organization than with variability-related measures. Rather than supporting a single dominant gait determinant, the results highlight a multidimensional pattern of gait–metabolic associations across multiple gait domains. These findings provide an empirical foundation for future studies seeking to better understand mobility performance and walking energetics in aging populations. Future work should determine whether interventions targeting gait speed, gait timing, and overall mobility function can improve walking efficiency and functional independence in later life.

## Figures and Tables

**Table 1:** Description of Gait Parameters according to functional profile.

	Parameter	Acronym	Total Sample	High Function (≥10 SPPB)	Low Function (<10 SPPB)		Parameter	Acronym	Total Sample	High Function (≥10 SPPB)	Low Function (<10 SPPB)
Spatial	Total Velocity (cm/s)	G1	85	53	32	Variability	Step Length SD (cm)	G18	85	53	32
	Stride Velocity (cm/s)	G2	85	53	32		Step Time SD (ms)	G19	85	53	32
	Step Length (cm)	G3	85	53	32		Stride Length SD (cm)	G20	85	53	32
	Stride Length (cm)	G4	79	50	29		Stride Time SD (ms)	G21	85	53	32
	Heel-to-heel (HH) Base Support (cm)	G5	85	53	32		Double Support Time SD (ms)	G22	85	53	32
	Toe In Out Angle (degrees)	G6	82	51	31		Single Support Time SD (ms)	G23	85	53	32
	Total Step Length Differential (cm)	G7	85	53	32		Swing Time SD (ms)	G24	85	53	32
Temporal	Total Cadence (steps/min)	G8	85	53	32		Stance Time SD (ms)	G25	85	53	32
	Cycle Time (ms)	G9	85	53	32		Support Base On SD (cm)	G26	85	53	32
	Step Time (ms)	G10	85	53	32		Stride Velocity SD (cm/s)	G27	85	53	32
	Double Support Time (ms)	G11	85	53	32		Heel Off On SD (ms)	G28	85	53	32
	Single Support Time (ms)	G12	85	53	32						
	Swing Time (ms)	G13	85	53	32						
	Heel Off On Time (ms)	G14	85	53	32						
	Stance Time (ms)	G15	85	53	32						
	Step Time Differential (ms)	G16	85	53	32						
	Total Cycle Time Differential (ms)	G17	85	53	32						

**Table 2:** Participants’ characteristics table.

Characteristic	Total	SPPB ≥ 10 (*N* = 53)	SPPB < 10 (*N* = 32)
Age (years)	73.69 ± 6.10	71.89 ± 5.79	76.69 ± 5.46
Gender	Male: 32 Female: 53	Male: 17 Female: 36	Male: 15 Female: 17
BMI (kg/m^2^)	27.90 ± 5.10	27.76 ± 5.15	28.13 ± 5.09
Gait speed (cm/s)	112.76 ± 21.36	121.69 ± 19.42	97.98 ± 15.56
SPPB score	9.95 ± 1.93	11.2± 0.9	7.8 ± 1.2
Below functional aerobic capacity threshold (Female < 18 mL/kg/min; Male < 20 mL/kg/min)	35 (41%) Female: 19, Male: 16	11 (21%) Female: 6, Male: 5	24 (75%) Female: 13, Male: 11

**Table 3:** Metabolic outcomes in older adults with high and low physical performance score according to SPPB.

Metabolic Parameter	Definition	Equation	Total Sample (*N* = 85)	High Function (≥10 SPPB, *n* = 53)	Low Function (<10 SPPB, *n* = 32)
Walk Percent of peak	Leisure walking metabolic rate normalized by peak aerobic capacity (VO_2_peak), expressed as a unitless ratio	$\frac{VO_{2,walk}}{VO_{2,peak}}\;$	0.57 ± 0.15	0.54 ± 0.15	0.61 ± 0.16
Walk V02 (mL/kg/min)	Steady-state VO_2_ at self-selected walking speed	$VO_{2,walk}$	10.74 ± 2.30	11.42 ± 2.36	9.62 ± 1.70
Gross*CoW* (mL/kg/m)	Total metabolic energy expenditure during walking	$\frac{VO_{2,walk}}{walking\;speed}$	0.20 ± 0.05	0.19 ± 0.04	0.22 ± 0.05
Reserve*CoW* (mL/kg/m)	Additional energy available per meter of travel before reaching maximal sustainable effort	$\frac{VO_{2,peak}-VO_{2,walk}}{walking\;speed}$	0.17 ± 0.09	0.18 ± 0.09	0.16 ± 0.09

**Table 4:** Gait parameters in older adults with high and low physical performance.

Parameter	Total Sample	High Function (≥10 SPPB)	Low Function (<10 SPPB)
Spatial			
Total Velocity (cm/s)	112.8 ± 21.4	121.7 ± 19.4	98.0 ± 15.6
Stride Velocity (cm/s)	113.4 ± 21.7	122.4 ± 19.8	98.5 ± 16.0
Step Length (cm)	61.8 ± 9.5	65.1 ± 8.9	56.4 ± 7.9
Stride Length (cm)	131.7 ± 18.0	136.5 ± 15.2	123.5 ± 19.7
Heel-to-heel (HH) Base Support (cm)	10.3 ± 3.2	9.4 ± 2.8	11.7 ± 3.4
Toe In Out Angle (degrees)	8.2 ± 4.1	8.0 ± 3.8	8.6 ± 4.6
Total Step Length Differential (cm)	2.6 ± 2.1	2.4 ± 2.0	3.0 ± 2.3
Temporal			
Total Cadence (steps/min)	109.2 ± 9.9	112.1 ± 9.0	104.4 ± 9.6
Cycle Time (ms)	1106.6 ± 102.2	1075.6 ± 88.1	1158.0 ± 104.3
Step Time (ms)	554.2 ± 51.0	538.7 ± 44.2	579.7 ± 51.8
Double Support Time (ms)	325.8 ± 61.4	304.6 ± 50.0	360.9 ± 63.0
Single Support Time (ms)	389.8 ± 31.1	384.9 ± 27.4	398.0 ± 35.5
Swing Time (ms)	389.8 ± 31.1	384.9 ± 27.4	398.0 ± 35.5
Heel Off On Time (ms)	77.9 ± 42.1	91.5 ± 36.0	55.3 ± 42.2
Stance Time (ms)	716.8 ± 78.2	690.7 ± 66.5	760.0 ± 77.9
Step Time Differential (ms)	19.6 ± 16.6	16.4 ± 7.5	25.1 ± 24.5
Total Cycle Time Differential (ms)	10.7 ± 6.6	11.7 ± 6.7	9.1 ± 6.2
Variability			
Step Length SD (cm)	1.9 ± 0.8	1.7 ± 0.7	2.3 ± 0.6
Step Time SD (ms)	17.7 ± 7.2	15.9 ± 6.5	20.8 ± 7.3
Stride Length SD (cm)	2.8 ± 1.2	2.5 ± 1.2	3.2 ± 0.9
Stride Time SD (ms)	23.8 ± 12.0	21.1 ± 10.0	28.4 ± 13.7
Double Support Time SD (ms)	18.9 ± 8.0	17.4 ± 7.3	21.4 ± 8.7
Single Support Time SD (ms)	15.8 ± 6.2	13.9 ± 4.6	18.8 ± 7.2
Swing Time SD (ms)	15.8 ± 6.2	13.9 ± 4.6	18.8 ± 7.2
Stance Time SD (ms)	21.3 ± 11.5	19.5 ± 9.6	24.4 ± 13.8
Support Base On SD (cm)	2.1 ± 0.9	2.0 ± 0.8	2.2 ± 1.1
Stride Velocity SD (cm/s)	3.4 ± 1.7	3.3 ± 1.7	3.4 ± 1.6
Heel Off On SD (ms)	28.6 ± 13.1	27.2 ± 12.9	31.0 ± 13.2

**Table 5:** Multivariable linear regression results for selected gait–metabolic pairs. All models adjusted for age, gender, and height.

Gait Feature	Outcome	Std β	95% CI	*p*-Value	Semi-Partial R^2^	Adj R^2^
Total Velocity (G1)	Preferred-speed metabolic rate (M2)	0.592	(0.381, 0.803)	<0.001	0.267	0.282
Stride Velocity (G2)	Preferred-speed metabolic rate (M2)	0.592	(0.381, 0.802)	<0.001	0.267	0.283
Step Length (G3)	Preferred-speed metabolic rate (M2)	0.476	(0.234, 0.718)	<0.001	0.153	0.163
Total Cadence (G8)	Preferred-speed metabolic rate (M2)	0.495	(0.282, 0.708)	<0.001	0.201	0.213
Cycle Time (G9)	Preferred-speed metabolic rate (M2)	−0.482	(−0.696, −0.269)	<0.001	0.192	0.204
Step Time (G10)	Preferred-speed metabolic rate (M2)	−0.482	(−0.696, −0.269)	<0.001	0.191	0.203
Double Support Time (G11)	Preferred-speed metabolic rate (M2)	−0.504	(−0.707, −0.301)	<0.001	0.222	0.236
Heel-Off On Time (G14)	Preferred-speed metabolic rate (M2)	0.339	(0.122, 0.556)	0.0026	0.103	0.110
Stance Time (G15)	Preferred-speed metabolic rate (M2)	–0.52	(−0.724, −0.309)	<0.001	0.22	0.236
Step Time SD (G19)	Gross cost of walking (M3)	0.286	(0.063, 0.508)	0.012	0.068	0.138

## Data Availability

Data available on request due to ethical reasons.

## Supplementary Materials

The following supporting information can be downloaded at: https://www.mdpi.com/article/10.3390/s26144543/s1, Table S1: Full correlation results between gait parameters and metabolic outcomes.

## Abbreviations

The following abbreviations are used in this manuscript:

SPPB	Short Physical Performance Battery
FDR	False discovery rate
