# Supplementary material for: Mapping the Multidimensional Link Between Spatiotemporal Gait Features and Metabolic Cost in Older Adults
Source: Sensors (Basel). 2026 Jul 17;26(14):4543. doi: 10.3390/s26144543 (PMC13431330; doi:10.3390/s26144543)
Supplement: Supplementary file 1 [file sensors-26-04543-s001.zip › sensors-4375092-supplementary.pdf]

sensors

MDPI

Table S1. Full correlation results between gait parameters and metabolic outcomes.

Gait Feature	Metabolic Feature	*N*	Pearson r	*p*-value	FDR q-value
G1	M1	85	−0.172	0.115	0.358
G1	M2	85	0.547	0.000	0.000
G1	M3	84	−0.231	0.035	0.184
G1	M4	84	0.045	0.687	0.906
G2	M1	85	−0.174	0.111	0.358
G2	M2	85	0.547	0.000	0.000
G2	M3	84	−0.229	0.036	0.185
G2	M4	84	0.049	0.657	0.876
G3	M1	85	−0.261	0.016	0.132
G3	M2	85	0.404	<0.001	0.002
G3	M3	84	−0.294	0.007	0.067
G3	M4	84	0.136	0.219	0.466
G4	M1	79	−0.028	0.807	0.911
G4	M2	79	0.260	0.021	0.146
G4	M3	78	−0.136	0.234	0.468
G4	M4	78	−0.027	0.813	0.911
G5	M1	85	0.110	0.317	0.546
G5	M2	85	−0.073	0.507	0.737
G5	M3	84	0.107	0.334	0.550
G5	M4	84	−0.119	0.281	0.533
G6	M1	82	−0.030	0.786	0.907
G6	M2	82	−0.154	0.168	0.446
G6	M3	81	0.059	0.602	0.812
G6	M4	81	0.042	0.712	0.906
G7	M1	85	−0.013	0.906	0.943
G7	M2	85	−0.013	0.909	0.943
G7	M3	84	0.111	0.315	0.546
G7	M4	84	0.064	0.560	0.794
G8	M1	85	0.101	0.359	0.567
G8	M2	85	0.464	<0.001	<0.001
G8	M3	84	0.031	0.777	0.906
G8	M4	84	−0.147	0.183	0.458
G9	M1	85	−0.098	0.375	0.583
G9	M2	85	−0.457	0.000	0.000
G9	M3	84	−0.014	0.897	0.943
G9	M4	84	0.145	0.189	0.460
G10	M1	85	−0.105	0.339	0.550
G10	M2	85	−0.456	<0.001	<0.001
G10	M3	84	−0.021	0.851	0.926
G10	M4	84	0.151	0.171	0.446
G11	M1	85	0.069	0.530	0.761
G11	M2	85	−0.506	<0.001	<0.001
G11	M3	84	0.116	0.292	0.544
G11	M4	84	0.003	0.979	0.988
G12	M1	85	−0.214	0.049	0.189
G12	M2	85	−0.257	0.018	0.132
G12	M3	84	−0.135	0.223	0.466
G12	M4	84	0.218	0.046	0.189
G13	M1	85	−0.214	0.049	0.189
G13	M2	85	−0.257	0.018	0.132
G13	M3	84	−0.135	0.223	0.466
G13	M4	84	0.218	0.046	0.189
G14	M1	85	−0.243	0.025	0.164
G14	M2	85	0.370	<0.001	0.006
G14	M3	84	−0.196	0.074	0.268
G14	M4	84	0.167	0.130	0.382
G15	M1	85	−0.043	0.696	0.906
G15	M2	85	−0.494	<0.001	<0.001
G15	M3	84	0.035	0.755	0.906

G15	M4	84	0.103	0.351	0.562
G16	M1	85	0.018	0.868	0.926
G16	M2	85	0.034	0.755	0.906
G16	M3	84	0.233	0.033	0.184
G16	M4	84	0.029	0.794	0.907
G17	M1	85	−0.138	0.208	0.466
G17	M2	85	0.090	0.411	0.622
G17	M3	84	−0.062	0.576	0.796
G17	M4	84	0.174	0.113	0.358
G18	M1	85	0.172	0.115	0.358
G18	M2	85	−0.111	0.313	0.546
G18	M3	84	0.170	0.122	0.370
G18	M4	84	−0.153	0.164	0.446
G19	M1	85	−0.010	0.927	0.952
G19	M2	85	−0.132	0.229	0.466
G19	M3	84	0.313	0.004	0.041
G19	M4	84	0.141	0.201	0.466
G20	M1	85	0.198	0.069	0.257
G20	M2	85	−0.134	0.221	0.466
G20	M3	84	0.085	0.440	0.657
G20	M4	84	−0.221	0.044	0.189
G21	M1	85	0.020	0.853	0.926
G21	M2	85	−0.078	0.479	0.706
G21	M3	84	0.267	0.014	0.132
G21	M4	84	0.112	0.309	0.546
G22	M1	85	0.145	0.184	0.458
G22	M2	85	−0.133	0.226	0.466
G22	M3	84	0.155	0.158	0.446
G22	M4	84	−0.095	0.392	0.602
G23	M1	85	−0.019	0.862	0.926
G23	M2	85	−0.222	0.041	0.189
G23	M3	84	0.235	0.031	0.184
G23	M4	84	0.108	0.327	0.546
G24	M1	85	−0.019	0.862	0.926
G24	M2	85	−0.222	0.041	0.189
G24	M3	84	0.235	0.031	0.184
G24	M4	84	0.108	0.327	0.546
G25	M1	85	−0.033	0.764	0.906
G25	M2	85	−0.003	0.980	0.988
G25	M3	84	0.152	0.167	0.446
G25	M4	84	0.109	0.325	0.546
G26	M1	85	−0.001	0.993	0.993
G26	M2	85	−0.034	0.754	0.906
G26	M3	84	−0.058	0.601	0.812
G26	M4	84	−0.035	0.752	0.906
G27	M1	85	0.120	0.275	0.533
G27	M2	85	0.136	0.216	0.466
G27	M3	84	0.032	0.773	0.906
G27	M4	84	−0.120	0.276	0.533
G28	M1	85	−0.037	0.736	0.906
G28	M2	85	−0.034	0.757	0.906
G28	M3	84	0.174	0.114	0.358
G28	M4	84	0.063	0.568	0.795
